# Creating pathways to just and sustainable food systems with citizen assemblies

**DOI:** 10.1080/13511610.2024.2309173

**Published:** 2024-01-31

**Authors:** Patricia Schmid, Léa Lamotte, Michael Curran, Sabin Bieri

**Affiliations:** a Centre for Development and Environment (CDE), University of Bern, Bern, Switzerland; b Institute of Geography (GIUB), University of Bern, Bern, Switzerland; c Department of Food System Sciences, Research Institute of Organic Agriculture FiBL, Frick, Switzerland

**Keywords:** deliberation process, citizen assemblies, just transformation, climate crisis, food systems, environmental justice

## Abstract

Food systems affect and are affected by the interrelated crises of climate change, biodiversity loss, resource depletion and health, amongst others. Transforming to sustainable approaches is vital, yet entangled with uncertainties, complexity and a great value diversion with stakeholders. Deliberative processes such as citizen assemblies offer a valuable contribution to such a transformation, since the crises and their responses affect everyday life, and therefore inviting individual and collective action. Still, who is included and whose knowledge counts affects outcomes. Theoretically anchored in concepts of environmental justice, our study analyses three nation-wide citizens’ assemblies on climate change and food systems from Western Europe. It assesses (a) how citizens’ assemblies can incorporate a broad set of viewpoints and design more substantive political answers to current crises, and (b) whether citizens’ assemblies include environmental justice aspects to facilitate social change. The paper argues that systematic and methodologically reflected inclusion of various positionalities can inspire decision-making processes in that they incorporate procedural, recognition, and distributional justice to address problems of climate change or modern food systems. It concludes with offering further approaches to include more than scientific knowledge in deliberative processes for a just transformation towards sustainability.

## Introduction

The crises our world faces today are unprecedented, interconnected and complex. The ongoing and accelerating crises of climate change, biodiversity loss, pollution and health, among others, endanger lives and well-being of current and future generations of humans and non-humans, and are unfairly distributed. The results mounting up from these crises, such as heatwaves, recent wildfires across Europe, and droughts, show the urgency for change.

The drastic effects of global crises can be exemplified by their interrelated links to food. The way we grow, distribute, access, prepare, eat and dispose of food affect and are affected by climate change, biodiversity loss, hunger and malnutrition, inequality, resource depletion and land degradation, among others (IPES-Food 2015). For instance, farming is vulnerable to the increased variability in precipitation and temperature, and agricultural fertilisers decrease agrobiodiversity (Vermeulen, Campbell, and Ingram 2012). Smallholder farmers, ethnic minorities, women and people with low economic resources are unequally exposed to extreme hazardous climate events or to daily pollution from air, water and soil (Schlosberg and Collins 2014). Environmental injustice, driven by racial discrimination and socioeconomical exclusion, is reinforced by climate change, which reflects and increases social inequalities (Mohai, Pellow, and Roberts 2009). Furthermore, just working conditions for all workers and beings involved across the food value chains and equality in economic and physical access to healthy, environmentally friendly and culturally appropriate food for all are not ensured by the dominant agro-industrial food system (Mohai, Pellow, and Roberts 2009). Various external shocks at the regional and international level, including the war in Ukraine and the Covid-19 pandemic, have exacerbated these inequalities (Fiske et al. 2022; Rabbi et al. 2023)

Business-as-usual is not an option in the face of the multidimensional crises of the global food system, whose processes, responses and outcomes are closely linked to social, economic and political mechanisms (Castree 2008). Characterised by large uncertainties, complexity and divergent values among stakeholders (Rittel and Webber 1974; Scoones et al. 2018), the global food system crisis can be identified as a ‘wicked’ problem. As current dominating production, distribution and consumption practices are the underlying causes of the issues, fundamental transformations are urgently needed. Yet there exist diverging views on how a food system transformation would look like, and what sustainability entails (Moragues-Faus and Marsden 2017). For example, technocratic and capital-intensive solutions and top-down approaches arguing for a green growth stand opposed to long-lived and new formations of community initiatives, subsistence practices and non-commodified relations with food (Moragues-Faus and Marsden 2017). As such, how to act is contested and leads increasingly to polarisation among political parties and citizens. The call for immediate change and transformation to sustainability (FAO 2022; HLPE 2020) conclude with the need to redesign agri-food policies based on democratic processes and call for methods that bring local voices into decision-making (HLPE 2020; Pimbert, Thompson, and Vorley 2001).

Existing political institutions, both by design and development, are unable to address the scale and urgency of these challenges. Political processes tend to be slow and indecisive in implementing substantial and radical changes. Further, political decisions are generally taken in the rhythm of the electoral cycle rather than in response to actual crises (Smith 2021). In response to the political deficit on immediate action, deliberative approaches have become more popular (Goodin and Dryzek 2006). This participatory turn signals a widespread acceptance of the need to include a range of social actors in more open policy forums to deal with increasingly complex issues (Blue 2015). Deliberative approaches are ‘grounded in an ideal in which people come together, based on the equal status and mutual respect, to discuss the political issues they face, and based on those discussions, decide on policies that will affect their lives’ (Bächtiger et al. 2018, 2). By evaluating new information and arguments made by other participants, they can make well-informed decisions and revise their preferences (Chambers 2003).

In Europe, the institutionalised deliberative wave has been building since the 1980s and became stronger in recent years (Chwalisz 2020). For instance, recent national citizen assemblies took place in Austria and the UK on climate change, and the first national citizen assembly on food system policy was carried out in Switzerland in 2022 (Buergerinnenrat 2023). The OECD report entitled ‘Catching the deliberative wave: Innovative Citizen Participation & New Democratic Institutions’ (Chwalisz 2020) is the first comprehensive body of work on the use and effectiveness of citizen assemblies and recommends deliberative processes for complex problems that involve trade-offs. It recommends them as a mean for better policy outcomesand enhance trust between citizens and government (Chwalisz 2020).

In this paper, we discuss the potential of citizen assemblies as a tool to respond to crises such as climate change or governing food systems, and how they incorporate justice aspects in their design and outcomes. To do this, we first articulate the contemporary food system crisis and citizen assemblies as a tool to articulate solutions, and put them in relation to environmental justice. Second, we conduct a comparative qualitative analysis of three citizen assemblies (two on climate change and one on food system policy) and assess the role of justice in their process design and recommendations. The paper concludes with a discussion of the key findings.

## Materials and methods

### Review of the literature on food systems and deliberative democracy

As a conceptual framework, we used an informal review of the literature. First, we discuss the necessary transformation of the global food system in crisis and highlight why justice is a central component towards transformation. Second, we connect key studies from the deliberative democracy literature that examine citizen assemblies with justice aspects lend from environmental justice. In both cases, we did not aim for a reproducible or systematic approach but selected key literature in the respective topics.

### Qualitative, comparative case study analysis

The paper employs a comparative case study approach based on the analysis of public material and scientific articles resulting from three European national citizen assemblies; two on climate change in Austria and the UK, and one on food system policy in Switzerland. The cases were selected based on their recent implementation, relevance to food systems, Western European context, national scale, and public availability of documentation and other material. While diverse forms of deliberative mini-publics occurred all over the world and at various scales (from local to global), we decided to engage only in a Western European context to better facilitate comparison within aligned conditions and a partly shared political system.

The material analysed consists of written, public documents from the citizen assemblies, related to their design and citizens’ recommendations. This approach focuses on the collective public statements and recommendations of the assemblies as a participatory learning and action approach (Kuruganti, Pimbert, and Wakeford 2008), in contrast to conducting individual interviews with participants or experts after the process.

The case study material was analysed using two analytic frameworks: (a) the OECD set of good practice criteria for deliberative processes (Chwalisz 2020) and (b) a perspective based on the three components (distribution, procedure and recognition) of environmental justice (Schlosberg 2007). These two frameworks were selected because of their relevance to evaluate the case studies in terms of their setup and outcomes. Additionally, the environmental justice framework plays well into some of the OECD criteria and complements with its specific justice focus. The following section explains these frameworks and relates them to the food system and citizen assemblies.

## Conceptual framework

### Transforming the global food system in crisis

Multiple socioeconomic and political drivers have pushed the food system into its current state, including large-scale commercial farming practices and long value chains that put profit over social and ecological well-being (Willett et al. 2019). The industrialisation, globalisation and corporatisation of the food system have been intensified by increasing financialisation (Clapp 2015). This resulted from liberalisation of agricultural futures markets in the 1980s and 1990s, leading to new speculative markets to emerge around agricultural commodities (e.g. ‘commodity index funds’) and direct investment in farmland (Clapp 2015). As a consequence, increasing institutional and corporate investments have spurred alarming trends in land and water grabbing, which increases asset prices and threatens local food security and nutrition, and has been observed across all of the world’s continents (Rulli, Saviori, and D’Odorico 2013). In terms of environmental impact, global agriculture is responsible for 21–37% of global emissions (IPCC 2019), of which the livestock sector alone is accountable for roughly 9% (Caro et al. 2014). Large-scale monocultures lead to soil depletion, and these effects as well as further influences from climate change impact farming (IPCC 2019). Additionally, agriculture uses 70% of freshwater resources in the world, mostly for irrigation (IPCC 2019). Dietary trends have seen rising meat consumption globally, along with other animal source protein (such as dairy, seafood), which put a huge pressure on life support systems (Godfray et al. 2018). Even though these impacts are severe, a third of the produced food is wasted along the food chain (IPCC 2019).

While the production of our daily food relies on the many smallholder farmers, the concentration of control by a handful of firms forms the decision-making (Pimbert, Thompson, and Vorley 2001). A handful of firms are involved and increasingly control what we eat from biotechnology to production and processing food (Pimbert, Thompson, and Vorley 2001). As a result, the food system resembles an hour-glass: at the top are millions of farmers and farm labourers who produce the food, and at the bottom are billions of consumers. At the narrow point in the middle are the few multinational corporations such as input suppliers, processors and retailers, which earn a significant profit from every transaction (Pimbert, Thompson, and Vorley 2001). These unjust power relations underline the lack of democracy in the global food system.

The social burden of these trends is immense and unjustly carried. Even though the narrative of the globalised food system promised to decrease hunger (Campbell 2013), hunger levels are rising again since 2014 while many suffer from diseases of malnutrition and overconsumption (FAO 2018). The 2022 Global Report on Food Crises (FAO 2022) highlights the remarkably high severity and numbers of people in crisis, driven by persistent conflict, pre-existing and COVID-19-related economic shocks, and weather extremes. Additionally, the war in Ukraine has led to global shortages of grains and fertilisers due to high import dependency and brittle globalized supply chains, particularly affecting farmers and consumers who cannot afford skyrocketing prices (IPES-Food 2020). Taken together, because of the inadequate nourishment of the world’s population and the many environmental systems and processes pushed beyond their safe boundaries by food production, a transformation of the food system is urgently needed (Willett et al. 2019).

For such a transformation, it is important to account that the crises do not affect all in the same way (Bailey 2011). As such, people who are currently more impacted due to unequal power relations need to be at the centre of restructuring to ensure a fair change. Some examples of the global food system inequalities include the structural hindrance for women to access land (Agarwal and Bina 1994; Meinzen-Dick et al. 2019); poor and toxic working conditions for mostly migrant workers along the food chain (Bogoeski 2022); erasure of traditional agricultural and spiritual knowledge relating to food preparation and consumption (Swiderska et al. 2022); and uneven economic and physical access to healthy and sustainable food (Sen 1982). Thus, the complex of racism, sexism and ethnocentrism continues to pervade all relations of domination (Vergès, 2019) and reinforces existing unequal power relations. If marginalised and previously excluded groups continue to be denied a seat at the table, the crises cannot be tackled in a just way. However, justice-relevant considerations in transforming the food system remain scare (Tribaldos and Kortetmäki 2022).

While these ‘wicked problems’ impact all citizens in one way or another, many consumers are estranged from them as well as from the realities on farms, as supermarkets display a variety of products whose sources are not easily recognisable. Yet, food is a basic human need, and the structure of the food system affects us all. The way food is produced and consumed forms part of everyday life and therefore inviting individual and collective action (United Nations 2019). As such, public deliberation on food systems offers a valuable contribution to address the crises.

### Citizen assemblies as a response to the food system crisis

Participatory tools such as citizen assemblies provide bottom-up, place-based answers that consider local needs, and diverse value positions. Citizen assemblies consist of a certain number of people who represent the population of a specific area in relation to age, gender, professions, political affiliations, among others, who then deliberate over a certain period of time on a given issue. Perhaps the best-known citizen assemblies and most effective in terms of policy implementation took place in Ireland on same-sex marriage (2015), and later on abortion and climate change (2016–2018). They not only led to changes in law, but also challenged the presumption that some obstacles to reform were immutable. During the COVID-19 pandemic, citizen assemblies formed in many European cities, such as the COVID-19 Forum in the German state of Saxony, or the Bristol Citizen’s Assembly on ‘ways out of the Covid-19 crisis’. On the issue of climate change, further local and national citizen assemblies took place in Austria, Denmark, Finland, France, Germany, Scotland and United Kingdom in 2020–2022. Additionally, a network was created to link existing initiatives and support further assemblies (KNOCA 2021).

The OECD developed principles for high-quality deliberative processes that result in useful recommendations for process design (Chwalisz 2020). The OECD report explored close to 300 deliberative processes, upon which the principles were based. The list of criteria, along with a description of how they were used in the case study research is provided in Table 1. These include the deliberation on a public problem; the commitment of commissioning authorities to respond to or act on recommendations in a timely manner; the constellation of participants as a microcosmos of the general public; efforts to ensure inclusiveness through remuneration and providing for care work; access to wide range of expertise and evidence; finding common ground through careful and active listening that weights and considers multiple perspectives (Chwalisz 2020, 17).

**Table 1.** Evaluation criteria for the three citizen assemblies compared in the study (based on Chwalisz 2020; Schlosberg 2007).

Criterion	Description
*OECD*	
Problem formulation	The task should be clearly defined as a question that is linked to a public problem.
Agreed political impact	The commissioning authority should publicly commit to responding to or acting on recommendations in a timely manner and should monitor and regularly report on the progress of their implementation.
Transparency and communication	Anyone should be able to easily find the following information about the process: its purpose, design, methodology, recruitment details, experts, recommendations, the authority’s response, and implementation follow-up. Better public communication should increase opportunities for public learning and encourage greater participation.
Demographic representation	Participants should be a microcosm of the general public; this can be achieved through random sampling from which a representative selection is made to ensure the group matches the community’s demographic profile.
Socio-economic inclusiveness	Efforts should be made to ensure inclusiveness, such as through remuneration, covering expenses, and/or providing/paying for childcare or eldercare.
Balanced, wide and partly open input material	Participants should have access to a wide range of accurate, relevant, and accessible evidence and expertise, and have the ability to request additional information.
High-quality facilitation	Group deliberation entails finding common ground; this requires careful and active listening, weighing and considering multiple perspectives, every participant having an opportunity to speak, a mix of formats, and skilled facilitation.
Sufficient depth and duration	For high-quality processes that result in informed recommendations, participants should meet for at least four full days in person, as deliberation requires adequate time for participants to learn, weigh evidence, and develop collective recommendations.
Independence of coordination team	To help ensure the integrity of the process, it should be run by an arm’s’ length co-ordinating team.
*Environmental Justice*	
Distribution	Allocation of benefits and burdens, focusing on recommendations
Procedure	Process of decision-making, fair inclusion of all involved people
Recognition	Recognition of certain groups or people, cultural aspects, recognised knowledges in both process and recommendations

### Justice in deliberative processes

Current power relations enforce environmental inequalities regarding race, ethnicity, gender, class, abilities and other social categories (Pulido 2017). These are often internalised and, as such, need to be reflected upon in participatory processes. To examine how these cases of citizen assemblies contribute to justice in social change, this analysis borrows from the three interrelated dimensions of environmental justice, being distributional justice, procedural justice and recognition (Schlosberg 2007). The early environmental justice scholarship, born out of a movement of racialised communities in North Carolina who lived close to hazardous waste sites (Mohai, Pellow, and Roberts 2009), focused mainly on distributional justice. The biological necessities for living a life towards flourishing should be justly accessed and enjoyed (Mohai, Pellow, and Roberts 2009). As such, all people should have the ability to breathe clean air, drink clean water, grow food within soil that is unpolluted or harm-filled, and eat fish, plants and animals without fear of poisoning (Stock and Szrot 2021). Later on, issues of representation, participation and self-determination formed part of the discourse (Young 1990), which formed into the framework with distributional, procedural and recognition justice (Schlosberg 2007). These three dimensions are subsequently put into relation to the design and outcomes of citizen assemblies.

Distributional justice looks at the allocation of benefits and burdens in a specific context or social system (Schlosberg 2007). As Smith (2003) points out, deliberation orients people toward the common good and makes space to consider the interests of future generations as well as non-human beings. As such, the crises can be considered with significance from short to long-term, counterbalancing the tendency in politics on the immediate (Willis, Curato, and Smith 2022). Citizen assemblies allow for recommendations less dependent on existing power structures (Dryzek et al. 2019). The variety of sources and evidence exposed to the assembly is a key factor, framing the issues and influencing both process and outcomes. As such, deliberative theory suggests that the issue frame should be a fair representation of conflicting views and arguments (Nabatchi et al. 2012, 210), which could help participants to reflect on how to equally (re)allocate wealth and responsibilities.

Procedural justice is concerned about the process of decision-making with a fair participation, equal say for involved people and transparent procedures (Fraser 2000). This concerns the process design such as voting mechanisms within citizen assemblies, and a facilitation that ensures that all voices are heard. Moral and ethical positions, the value of knowledges of different actors, particularly the most affected and vulnerable, is considered in deliberative democracy literature (Hammond 2020). Deliberative democracy, at its heart, aims to counter the expression of dominant power and interests for their own benefit (Willis, Curato, and Smith 2022). It opens a space for collective decision-making within a range of perspectives that might otherwise be absent from expert communities and political or media discourses (Blue 2015). Additionally, participants may feel pressured that the recommendations are accepted by the broader public and policy makers and thus soften their demands. These inherent power relations are crucial to consider for the process design and, as stated by Curato (2019, vi), deliberation ‘gains relevance when it navigates complex relations of power in modern societies, learns from its mistakes, and remains epistemically humble but not politically meek’.

Finally, recognition deals with practices of cultural domination, non-recognition rendering certain peoples or groups invisible, and disrespect of certain peoples or groups in public and cultural discourse (Fraser 2000). Recognition valorises capabilities, experiences, contributions, knowledges and normative judgement (Fraser 2000) of the participants. Further, scholars have discussed knowledge representation in citizen assemblies (Blue 2015; Petts and Brooks 2006) and whether lay publics are viewed as lacking requisite knowledge who need to be informed to take good decisions. Such a bias towards expert knowledge can hinder the inclusion of other knowledges (Blue 2015). Iris Young’s conception of communicative democracy aims to include diverse knowledges within deliberation with

particularities instead of universalisms, narratives instead of speeches, emotional components instead of abstract reason, bodily expressions instead of protocols and formalism, thus making pluralism and its diverse forms of evidence emerge, so that people and groups previously silenced and obliterated can feel part of the democratic dynamics. (Lima and Sobottka 2020)

The importance of the positionalities and knowledges of citizens can be exemplified through food. All of us need to eat to live and should therefore have a say in the access to food that is healthy, culturally appropriate and respectful of the environment. Thus, a representative sample of the population automatically includes farmers, farm workers, distributors and consumers, who can share their perspectives in a citizen assembly.

To understand the procedure and how citizen assemblies contribute to environmental justice in an European setting, the paper subsequently presents the analysis of three country-wide citizen assemblies in terms of the procedure and recommendations and therein incorporation of the mentioned justice aspects.

## Results

### Case studies: citizen assemblies as response to systemic crises

In this section, the three selected citizen assemblies are explored in terms of process and outcomes. A summary of characteristics and evaluation results is provided in Table 2.

**Table 2.** Summary of the characteristics and evaluation of each case study with respect to the OECD criteria for good practice deliberation.

Case study and country	Der Klimarat (climate assembly), AU	Climate assembly, UK	Citizen assembly on food system policy, CH
**Schedule**	2022 (6 months)	2020 (5 months)	2022 (6 months)
**Problem formulation**	Climate policy up to 2040 (general task: ‘What do we have to do today, to live in a climate friendly future tomorrow?’)	Climate policy up to 2050 (specific task: ‘How should the UK meet its target of net zero greenhouse gas emissions by 2050?’)	Food system policy up to 2030 (specific task: ‘How should a food policy for Switzerland look like that makes healthy, sustainable, animal-friendly and fairly produced food available to all people by 2030?’)
**Agreed political impact**	No direct policy consequences. Federal council promised to deal with recommendations.	No direct policy consequences.	No direct policy consequences. Federal council promised to deal with recommendations.
**Transparency and communication**	Information and details of process and outcomes are readily available	Information and details of process and outcomes are readily available	Information and details of process and outcomes are readily available
**Demographic representation**	Random selection according to characteristics of age, gender, highest completed school education, income, regions, country of birth, degree of urbanisation, to represent people living in Austria	Sortition according to age, gender, educational qualification, ethnicity, where in the UK they live, whether they live in an urban or rural area, and attitudes to climate change	Random selection representative of the Swiss resident population in terms of age, gender, language, place of residence, and political interests.
**Socio-economic inclusiveness**	Small renumeration for participants, travel, food, and accommodation expenses during meetings.	Small renumeration for participants, travel, food, and accommodation expenses during meetings. Costs related to childcare or bringing a carer covered.	Small renumeration for participants, travel, food, and accommodation expenses during meetings.
**Balanced, wide and partly open input material**	Mixed consortium of scientific experts and interest groups	Mixed consortium of scientific experts	Mixed consortium of scientific experts and interest groups
**High-quality facilitation**	Independent facilitation and mixed format of inputs, discussions	Independent facilitation and mixed format of inputs, discussions, preferred visions	Independent facilitation and mixed format of inputs, discussions, learning journeys
**Sufficient depth and duration**	Six full weekend meetings (all present) during 6 months	Six full weekends, three present and three online during 5 months	Two full weekends to start and close, one day mid-meeting (all present); six online meetings; 2 learning journeys per participant. During 6 months
**Independence of coordination team**	Commissioned by Federal Ministry for Climate Protection, Environment, Energy, Mobility, Innovation and Technology, participant recruiting by Austria’s Federal Statistical Office, facilitation by consortium of ÖGUT, pulswerk und PlanSinn	Commissioned by six select committees of the House of Commons, participant recruiting by Sortition Foundation, facilitation by The Involve Foundation, branding and website by mySociety	Commissioned by grassroot organisation ‘Landwirtschaft mit Zukunft’, the foundation ‘Biovision’ and the SDSN network for sustainability solutions, participant recruiting by Demoscope, facilitation by Collaborato Helvetica

#### Case 1: Der Klimarat (climate council) Austria

At the end of 2021, 58% of the population in Austria were of the opinion that the political system does not function well (SORA, 2021). To respond to a popular petition claiming for a climate referendum which gathered 400,000 signatories (Verein Klimavolksbegehren 2021), the Federal Ministry for Climate Protection, Environment, Energy, Mobility, Innovation and Technology commissioned the creation ‘der Klimarat’, the first citizens’ council at the federal level. This national citizens’ assembly was formed to explore new political avenues for addressing climate change, debate policy responses and guide future good practices to achieve Austria’s climate targets (Buzogány et al. 2022; Hollaus 2022). From January to June 2022, 84 people, randomly selected though Austria’s Federal Statistical Office according to characteristics of age, gender, highest completed school education, income, regions, country of birth and degree of urbanisation, to represent people living in Austria, were tasked with drafting measures to submit to National Council and the Federal Ministry for Climate Protection (Hollaus 2022). Propositions should answer the open question: ‘What do we need to do today to live in a climate-friendly future tomorrow?’ The timeline was further specified up to 2040.

According to the resolution of the Austrian National Council, the Climate Council was a ‘participatory process for discussing and elaboration of concrete proposals for the climate protection measures necessary to achieve the goals on the way to climate neutrality in 2040’. (ARGE Klimarat, 2022, 11). At its core laid the question of how to make thoughtful decisions that combat climate change and empower citizens simultaneously (Buzogány et al. 2022). On the first weekend, the Federal Council guaranteed the participants that they would deal with each of the Climate Council’s proposals (ARGE Klimarat, 2022:31), yet did not specify how. The measures were submitted to the Climate Cabinet and the Federal Government respectively with a press conference. As such, the final decision on the implementation of the proposed measures were in the hands of the political authorities (Hollaus 2022). The participating citizens decided to set up an association after the process and regularly meet with organisations and politicians to further the demands of the citizen assembly (Noren 2022).

The evaluation of Buzogány et al. (2022) highlights an ‘adequate level of knowledge integration’. A mixed consortium of scientific experts provided participants with information texts and video interviews. During the first three weekends, scientists gave inputs to the five defined fields of action, being Energy, Production/Consumption, Food/Land Use, Mobility and Housing. The task was discussed in ten smaller groups, consisting of two groups within each of the five themes. The group met physically on six full weekends and prepared individually during the time in-between. The scientific consortium compiled four to five levers per theme, and the ten working groups developed recommendations on these levers. In their analysis, Buzogány et al. (2022) also show that the deliberative process succeeded in bringing together participants from different social groups and encouraged them to engage in dialogue and self-reflection.

The climate assembly agreed on a total of 93 recommendations within the five themes (ARGE Klimarat, 2022). The measures are very diverse and operate at different levels of government from local to national and involve different departments. The spectrum ranges from compulsory climate change education in schools to the introduction of a carbon tax of 55€/t CO_2_, rising to 240€/t by 2030, with specific social design, to a ban on the destruction of new goods and services (Clar, Omann, and Scherhaufer 2022). As such, the recommendations call for a more demanding climate policy than existing Austrian policy-making in the field (Buzogány et al. 2022).

Justice in terms of distribution has been mentioned in the recommendations. The preface of the recommendations outlines that ‘[w]e know that there is a danger that the socially weakest part of the population will have to bear a particularly high burden in terms of climate protection, which is why the proposals also take into account that compensation must be provided here, e.g. through an urgently needed tax reform’ (ARGE Klimarat 2022, 7). Furthermore, the council elaborated on principles for political action, which includes regulations and framework conditions that make climate-friendly action very simple for everyone; that climate protection should not be a luxury; that climate protection measures must not lead to socially weaker groups losing out further, thus attention must be paid to social balance; people with a higher income should make a higher contribution to climate protection; the forming of cross-border alliances and financial and know-how support for other countries; institutionalising citizen participation; and the regular evaluation and adaption of measures and strategies. These principles were of a general nature. In specific recommendations, distributional justice was considered in two recommendations, namely introducing greenhouse gas tariffs and providing funding for innovative distribution channels. In terms of greenhouse gas tariffs, food and agricultural products should be subject to a greenhouse gas tax. As higher food prices due to these taxes are an additional burden on low-income households, a redistribution should compensate for it, as climate protection should not depend on income (ARGE Klimarat 2022, 47). In terms of funding for innovative distribution channels, the recommendation highlighted that the security of supply and social compatibility are not neglected. Social compatibility is currently also impaired by overcapacities that are exported cheaply to countries in the global South and destroy local economic structures there. According to the recommendations, these exports through overproduction should be avoided in the future by moving away from the current support system towards an incentive and support system that is more focused on climate protection and other social needs (ARGE Klimarat 2022). Thus, a fair distribution is especially discussed within economic means.

#### Case 2: Climate assembly UK

As the first nation-wide citizen assembly in the United Kingdom, the UK climate assembly took place between January and May 2020. The assembly was commissioned by the UK Parliament in autumn 2019 (Elstub et al. 2021) by six committees of the House of Commons (Business Energy and Industrial Strategy; Environmental Audit; Housing, Communities and Local Government, Science and Technology; Transport; and Treasury) (UK Parliament 2023). The discussion question was formulated as ‘How should the UK meet its target of net zero greenhouse gas emissions by 2050?’ (UK Parliament 2023).

108 people took part in the climate assembly, and facilitation was provided by an independent organisation. Three organisations were contracted to run the climate assembly, one of which independently recruited the members through sortition. Members were represented according to age, gender, educational qualification, ethnicity, UK place of residence, urban or rural setting, and attitudes to climate change, to represent the UK population according to recent statistics of these metrics (Climate Assembly UK 2020). Inclusiveness was aspired with a small renumeration for participants, and travel, food, and hotel expenses.

The parliament showed a strong interest in citizen’s opinion, but did not commit to implement any of the recommendations. In comparison to the Austrian case, the UK government set a legally binding target of net zero greenhouse gas emissions by 2050, and the citizen assembly was initiated to consider how this could be achieved. The resulting recommendations should be used by politicians and policy makers to scrutinise Government’s policies and inform on public preferences. Ten areas were covered in the discussions: underpinning principles for the path to net zero; travel on land; travel by air; heat and energy use at home; eating and the use of land; buying; electricity; greenhouse gas removal; Covid-19; and additional recommendations. The final report was presented to the six select committees in September 2020.

On all six weekends, assembly participants received inputs from stakeholders and researchers and discussed specific topics related to reducing emissions. There was not a final voting on recommendations, as they were jointly discussed, and individual concerns were captured in the final report. Half of the meetings took place in person and had to be moved to online discussions due to COVID-19 restrictions. The climate assembly came forward with over 50 recommendations in a detailed 556-page report (Climate Assembly UK 2020). The evaluation by Elstub et al. (2021) highlights that the participants became more knowledgeable on climate change and that their conviction on the achievability of net zero greenhouse gas emissions target evolved through the process. The researcher team of the climate assembly concluded that the involved citizens generated far more ambitious policies than politicians have ever come up with (Mellier & Wilson 2020). Nevertheless, Elstub et al. (2021) showed in their evaluation of the deliberative process that the effects of the process are endangered by the length of the assembly report, the lack of public awareness of the process and the turnover in the commissioning members.

In the introduction section of the report, fairness was a key theme:

as with most things in life, the solutions to climate change are neither easy nor free, but they need to be fair. Fair to people with jobs in different sectors. Fair to people with different incomes, travel preferences and housing arrangements. Fair to people who live in different parts of the UK. (Climate Assembly UK 2020, 6)

‘Fairness within the UK, including for the most vulnerable (affordability, jobs, UK regions, incentives and rewards) in actions, not just words’ (Climate Assembly UK 2020, 12) was the second highest principle ordered in priority, just after education. Fairness for the most vulnerable globally was also a principle. This implies a broad understanding of fairness as it does not specifically mention intersectional or marginalised groups, except for ‘most vulnerable globally’. In specific recommendations, making low-carbon food more affordable was a recommendation that took distributional justice into account with ‘[s]upport for people on low incomes to be able to access and cook/use healthy local foods’ (Climate Assembly UK 2020, 263). Other aspects of distributional justice were not reflected in the recommendations.

#### Case 3: Swiss citizen assembly on food system policy

The first national citizen assembly in Switzerland took place over six months from June to November 2022. The task was clearly formulated as a question linked to a public problem: ‘How should a food policy for Switzerland look like that makes healthy, sustainable, animal-friendly and fairly produced food available to all people by 2030?’

The commissioning authority consisted of a grassroot organisation ‘Landwirtschaft mit Zukunft’ (www.landwirtschaftmitzukunft.ch), the foundation ‘Biovision’ (www.biovision.ch) and the Sustainable Development Solutions Network (SDSN; https://sdsn.ch) and it was financially supported by federal state’s offices for Agriculture, Food Safety and Veterinary Affairs and for the Environment (Buergerinnenrat 2023). Such an institutional support legitimized the deliberative process. The assembly participants consisted of 80 people, recruited through a random process by an independent market research company, to be as representative as possible of the Swiss resident population in terms of age, gender, language, place of residence and political interests. Inclusiveness was aspired with a small renumeration for participants, travel and hotel expenses if necessary, and solutions for special assistance or care work were sought after. However, there were no general child care or other care services available. The assembly was conducted in German, French and Italian with translations where necessary, so that citizens from all parts of Switzerland could participate and exchange without limits of national languages.

An exchange event was organised between the citizen assembly, members of the cantonal and national council, and scientific members at the end of the process in November 2022. Additionally, during a one-day event at the beginning of February 2023, the recommendations were officially handed over to the federal council and further discussed between representatives of the jury, scientific members of the SDSN network, parliament members and other stakeholders. Thus, the government showed interest in the process, yet did not commit to implement the recommendations.

The guiding question was discussed in ten smaller groups, consisting of two groups within five themes: health, environment, social, economy and production. Facilitation was conducted through an independent organisation focusing on facilitating various change processes. The discussion in smaller groups aimed for all participants to share their voices. There were five full-day in-person meetings and six online meetings. The starting weekend to get to know each other, understand the process and receive inputs from science and a diverse range of stakeholders and interest groups; a mid-term day to discuss ideas and recommendations across smaller groups; and a finalising weekend to complete recommendations and vote on them in plenary. Six online meetings of two hours each were held in-between, focusing on dialogue in the respective groups per theme. Additionally, the participants joined two organised day excursions to actors that are creating change within the food system. During these learning journeys to a variety of innovative farms, producers and processors, participants learned from the knowledge of diverse actors. As such, a range of knowledge and inputs was given to participants, while they were informed that stakeholders represent a certain position and should be viewed as such (Buergerinnenrat 2023).

A total of 126 recommendations were developed and accepted during the final voting day. They encompassed concrete and detailed ideas along the themes of ameliorating the social and economic situation of farmers, align agricultural practice and subsidies towards sustainability, align processing and trade, political instruments as well as education, consulting, and research towards sustainability, raising awareness towards health and sustainability for consumers, promote the truth of cost, and promoting the marketing of healthy and sustainable products (Buergerinnenrat 2023).

In terms of distributional justice, the recommendations addressed producers or consumers in a uniform way without intersectional distinctions. We identified four recommendations towards more specific distributional justice. First, the recommendation 85 concerning justice in food chains aims to align labels with social aspects in terms of the responsibility of corporations towards working conditions in their supply chains. Second, the recommendation 92 on price suggests to lower prices for healthy and sustainable products, so that people with lower incomes can eat healthy and seasonal. Third, the recommendation 93 on redistribution of profit voices for a fair payment of workers and actors in supply chains, as well as for redistributing profit from unsustainable products towards poorly paid actors in supply chains. Fourth, the recommendation 109 suggests providing grocery vouchers for healthy and sustainable food for people with less economic means.

## Discussion

The three cases all incorporate the best practices as suggested by OECD with a slight variability depending on their context. The setup of the three case studies includes a fair representation of inhabitants of each country with slightly differing criteria. In all cases, attention was given to inclusion in terms of financial remuneration, yet no specific care facilities were mentioned. The independent and professional facilitation increased that all voices were heared. In terms of outcomes, all three examples shows that citizens can put forward concrete recommendations on pressing issues and demand action that go further than governments’ agendas. This shows that citizens support a radical change when they are involved with the topic, which, in turn, could motivate policy makers to implement more profound measures than currently. In the case of the Austrian climate assembly, the provincial councillor of Salzburg voiced that ‘[t]here is no lack of knowledge about what to do. We do not need new papers, not more concept writers. We need encouragers and courage for concrete results’ (ARGE Klimarat 2022, 35).

The three cases have shown that community interests and needs of marginalised people are considered in such deliberations, but are not explored in-depth.The statements of including the most vulnerable in the UK case highlights the participants’ understanding that climate change is embedded in complex international dynamics, yet measures remain on the surface. In Austria, the distributional justice perspective focused on social compatibility, and overcapacities that are exported cheaply to countries in the global South, thus reinforcing power imbalances. The report from the Klimarat suggested that such exports through overproduction should be avoided in the future by moving away from the current support system towards an incentive system that is more focused on climate protection and other social needs (ARGE Klimarat 2022, 47). The Swiss case explicitly mentioned justice in only four recommendations, which are, like the UK case, focusing on economic redistribution. As such, there remains a lack of fully incorporating distributional justice in the recommendations.

In terms of representation justice, participants should be able to voice their opinions, experiences, hopes and concerns. This was possible through independent facilitation, where all three cases mention the importance of hearing all voices. At the same time, all three cases are strongly oriented on scientific inputs, especially during the introductory meetings. The UK climate assembly, for instance, began with expert leads deciding on ‘the range of evidence the assembly would need to hear to ensure members heard a balanced, accurate and comprehensive view of the topic’ (Climate Assembly UK 2020), which makes the people’s own experience secondary to expert inputs. While the cases gave attention to provide a range of views and inputs, more recognition and dedicated time could be given to citizens’ own knowledges.

Thus, a suggestion is the inclusion of more diverse knowledges in these processes. The Swiss citizen assembly included for example two field visits per participant to a diversity of food-related initiatives, which allows for a different way of learning from concrete experiences and includes a variety of knowledges. Inspiration could also come from other deliberation processes, for example through the KNOCA network in Europe. It may be valuable to try out a format where citizens are included in shaping the agenda, for example, the themes to discuss. (Pimbert, Thompson, and Vorley 2001). Other important factors are culture and emotional values connected to the problem that would allow for other, intimate knowledge to emerge as an addition to scientific inputs. To do so, Iris Young (2001) advocates for various articulations of reason-giving, such as storytelling or testimonies, or Gordon, Haas, and Michelson (2017), advocate for role-playing games in deliberative processes.

In terms of the process frame, Stirling (2008) offers the distinction between opening up and closing down of policy options in deliberative processes. Processes that follow a closing-down approach show a set of possible pathways that make sense under a particular framing condition. The example from the UK climate assembly illustrates this pre-defined approach well. Here, participants deliberated on pre-defined pathways, while, in the other two cases, citizens brought up their own options. The opening up process, as such, examines neglected issues, marginalised perspectives and ignored uncertainties. The outputs are more of exploratory nature and can accommodate a broader range of options.

A further crucial point within a well-structured and inclusive deliberation process is that participants acquire skills of deep listening and learning from other perspectives. Involved citizen might also want to remain actively engaged with the topic, as the Austrian case shows with the establishment of an association of participants of the citizen assembly.

Finally, there still exists an implementation gap of the recommendations. Indeed, in all three cases, the recommendations were read with interest by political institutions, yet not concretely implemented. The long-term impact of citizen assemblies depends on the commitment of policy makers to take up the recommendations. Without it, citizen assemblies’ voices might be noted, yet established political and institutional power will remain untouched. As public authorities from all levels of government increasingly show their interest in citizen assemblies and other representative deliberative processes to tackle complex problems, they should show more commitment as to reinvigorate and enhance trust from citizens.

## Conclusion

This paper discussed citizen assemblies as a tool to find solutions to current crises and advance towards transformation. It framed the global food system in crisis and how deliberative approaches could, with an integration of justice, provide necessary recommendations. The three case studies of national citizen assemblies in Austria, the United Kingdom and Switzerland showed that well-organised citizen assemblies can bring forward a variety of voices and opinions and that their recommendations go further than existing policies. The OECD good practice criteria for deliberative processes are valuable for an institutional and thorough setup, however, justice aspects should be better integrated. The environmental justice framework with distributional justice, procedural justice and recognition provides a helpful approach to ensure that justice is incorporated in the design and outcome of deliberative approaches.

As a shortcoming, we find that the investigated cases tend to focus on scientific inputs rather than participants’ own experiences and knowledges. It is crucial to reflect on whose narrative is given space, taken up and seen as valid in deliberative processes. This has somewhat been taken up within the deliberative processes design, yet, especially when deliberating on topics such as food where everyone is part of the chain, more importance could be laid on participants’ sources. This may be aided through testimonies or games.

Further, we found that justice is not an explicit concept used both in the structure of the researched citizen assemblies and their recommendations. We argue that their outcomes would benefit from such an integration. While justice aspects came up within the recommendations, they remained rather on the surface with speaking of a homogenous group of vulnerable people, and remained scarce in the number of recommendations.

Moreover, we framed citizen assemblies as a tool to strengthen policies and enhance trust between government and citizens. Yet, governments and policy makers did not oblige to implement the recommendations and continue to be slow in advancing measures for change. As such, the assemblies have not effectively been used for this goal.

We conclude that citizen assemblies could play a major role in finding solutions to crises and complex issues, if distributional, procedural and recognition justice form a part of the framework, participants’ knowledges are given more attention, and governments commit to the results.
